# Structural transformation and phase change properties of Se substituted GeTe

**DOI:** 10.1038/s41598-021-87206-x

**Published:** 2021-04-07

**Authors:** Roopali Shekhawat, Haritha Pamuluri, Vinod Erkkara Madhavan, K. Ramesh

**Affiliations:** Department of Physics, Indian Institute of Science, Bengaluru, 560012 India

**Keywords:** Energy science and technology, Materials science, Physics

## Abstract

GeTe_1−x_Se_x_ (0 ≤ x ≤ 1.0) alloys have been prepared both in bulk and thin film forms to study the effect of selenium (Se) substitution for tellurium (Te) on the phase change properties. It is observed that with increasing Se substitution in GeTe, the structure transforms from rhombohdral structure to orthorhombic structure. Rietveld Refinement analysis support the phase transformation and show that the short and long bond lengths in crystalline GeTe decrease with increasing Se substitution but the rate of reduction of shorter bond length is more than the longer bond length. The GeTe_1−x_Se_x_ thin films undergo amorphous to crystalline phase change when annealed at high temperatures. The transition temperature shows an increasing trend with the Se substitution. The contrast in electrical resistivity between the amorphous and crystalline states is 10^4^ for GeTe, and with the Se substitution, the contrast increases considerably to 10^6^ for GeTe_0.5_Se_0.5_. Devices fabricated with thin films show that the threshold current decreases with the Se substitution indicating a reduction in the power required for WRITE operation. The present study shows that the crystalline structure, resistance, bandgap, transition temperature and threshold voltage of GeTe can be effectively controlled and tuned by the substitution of Te by Se, which is conducive for phase change memory applications.

## Introduction

GeTe (Group IV–VI) based crystalline and amorphous materials find a wide range of applications in many fields^1–7^. Under the influence of electric and optical fields, GeTe has shown rapid and reversible transition between the amorphous and crystalline states, a prerequisite for the phase change memory (PCM) device applications. An ideal PCM material should possess fast crystallization speed, high thermal stability, endurance, and scalability^5^. Also the PCM material should not undergo phase separation during crystallization. In materials showing phase separation, crystallization is accompanied by diffusion of atoms, which is a slow process and limits the crystallization speed^8^. The well-known PCM material Ge_2_Sb_2_Te_5_ (GST) lies in the GeTe-Sb_2_Te_3_ pseudo-binary tie-line, where one end of the tie-line is GeTe material^6^. GST has been of interest for optical storage applications^9^, but it has not been ideal for phase-change random access memory (PCRAM) applications due to its low crystallization temperature (Tc), poor data retention ability^10^ and low resistance contrast. Se is a smaller and more covalent atom compared to Te and it is found that Se doping enhances the phase change properties of GST^11–15^. There are reports that Se alloys such as Ga-Sb–Se, Sb–Se possess higher Tc, better data retention, higher switching speed, lower thermal conductivity^16^, and lower melting temperature with respect to GST. However, the resistance ratio is limited to about 3-orders of magnitude^5,17^. Any efforts to enhance this contrast would be highly desirable to realize the multi-level storage in PCM devices. GeTe has emerged as an alternative due to its comparatively improved Tc, data retention^18^ and resistance contrast capabilities. In crystalline phase GeTe material occur in two structures i.e. rhombohedral structure and cubic NaCl structure, with 10% of vacancies occurring at Ge sites^18^. In the same Group IV-VI materials, crystalline GeSe has a orthorhombic structure^19–21^. Owing to the large solubility of GeSe in GeTe^22^, we propose the composition GeTe_1−x_Se_x_ (0, 0.05, 0.25, 0.50, 0.75, 0.875, 1.0) covering all ranges to study the effect of Se substitution for Te in GeTe alloy to further improve its phase change properties. It would guide and explore the usefulness of various Se substituted GeTe material for PCRAM applications.

## Results and discussion

**Figure 1:**
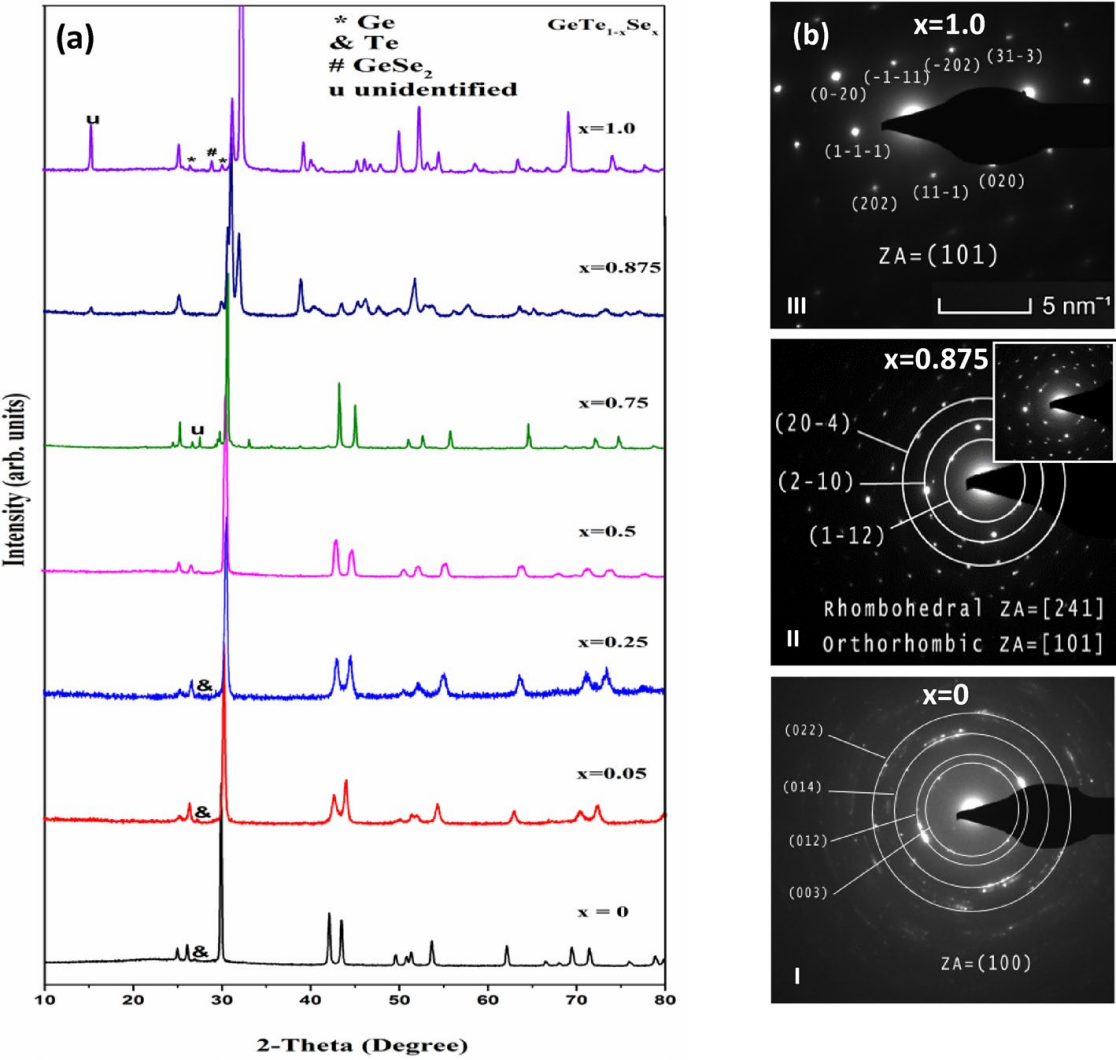
(**a**) XRD patterns of melt quenched GeTe_1−x_Se_x_ bulk samples 0 ≤ x ≤ 1.0. For x ≤ 0.75, the structure follows the rhombohedral and for x > 0.75, the structure changes to Orthorhombic. x = 0.875 sample shows mixed phases of both rhombohedral and orthorhombic structure. (**b**) Diffraction patterns of x = 0, 0.875 and 1.0, showing structural transition (I-III) complementary to XRD patterns. Inset in II is showing circular rings pattern with spot pattern indicating mixed phases.

XRD pattern of the bulk GeTe_1−x_Se_x_ is shown in Fig. 1 which demonstrates the effect of the Se substitution for Te. For GeTe (x = 0), diffraction peaks match precisely with the standard pattern of ICSD^23^ having rhombohedral structure with minute amount of phase segregated elemental Te. From Fig. 1, it is observed that the structure remains unchanged upto x = 0.75. The effect of Se substitution is seen as the broadening in peak width and shift of peak positions to higher 2-theta values. The increase in the width of the peaks indicates that the increase of Se showing distortion in the rhombohedral lattice. The shift of the peaks to higher 2-theta values is due to the compression of lattice dimensions upon the substitution of smaller Se atom for Te. For x > 0.75, the structure gradually changes towards orthorhombic structure. It can be observed that, for x = 0.875, the XRD shows the mixture of both rhombohedral and orthorhombic phases indicating the solubility limit of GeSe in GeTe is about 75% (x = 0.75). The XRD pattern of GeSe (x = 1.0) shows orthorhombic phase with some minor peaks from the cubic Ge and GeSe_2_ phase^24^.

Te, a giant atom (142 pm), Influences the nearby atoms to a larger extent compared to the smaller Se atom (119 pm). Electro-negativity of Se (2.55) is higher than Te (2.1) on the Pauling scale. When Te is substituted with Se, higher energy Ge–Se bonds (2.14 eV) are formed at the expense of low energy Ge–Te bonds (1.53 eV) owing to higher electronegativity of Se. The bond length of Ge–Se (~ 2.53 Å) is also short compared to the bond lengths of Ge–Te (2.83 and 3.16 Å). Increase of the stronger Ge–Se bonds and the decrease of the weaker Ge–Te bonds increase the rigidity of the structural network. These effects collectively result in the reduction of the lattice parameters and influences the properties for the Se substituted GeTe samples.

Transmission electron microscopy (TEM) was used to capture the microstructure of x = 0, 0.875 and 1.0 bulk samples to complement the XRD results. The structure transformation from rhombohedral to orthorhombic structure with increasing Se subtitution in GeTe samples is shown in Selected Area Electron Diffraction (SAED) pattern in Fig. 1b. The ring pattern in Fig. 1b (I) indicates the polycrystalline nature of x = 0 sample and the pattern is identified and indexed for rhombohedral crystal structure (zone axis (ZA) [100]). While the SAED pattern of x = 1.0 (Fig. 1b (III)) shows spot pattern and is indexed for orthorhombic crystal structure (ZA [101]). Figure 1b (II) for x = 0.875 showing both ring and spot patterns, from rhombohedral and orthorhombic phases respectively, indicating the mixture of two phases, in accordance with XRD results.

**Figure 2:**
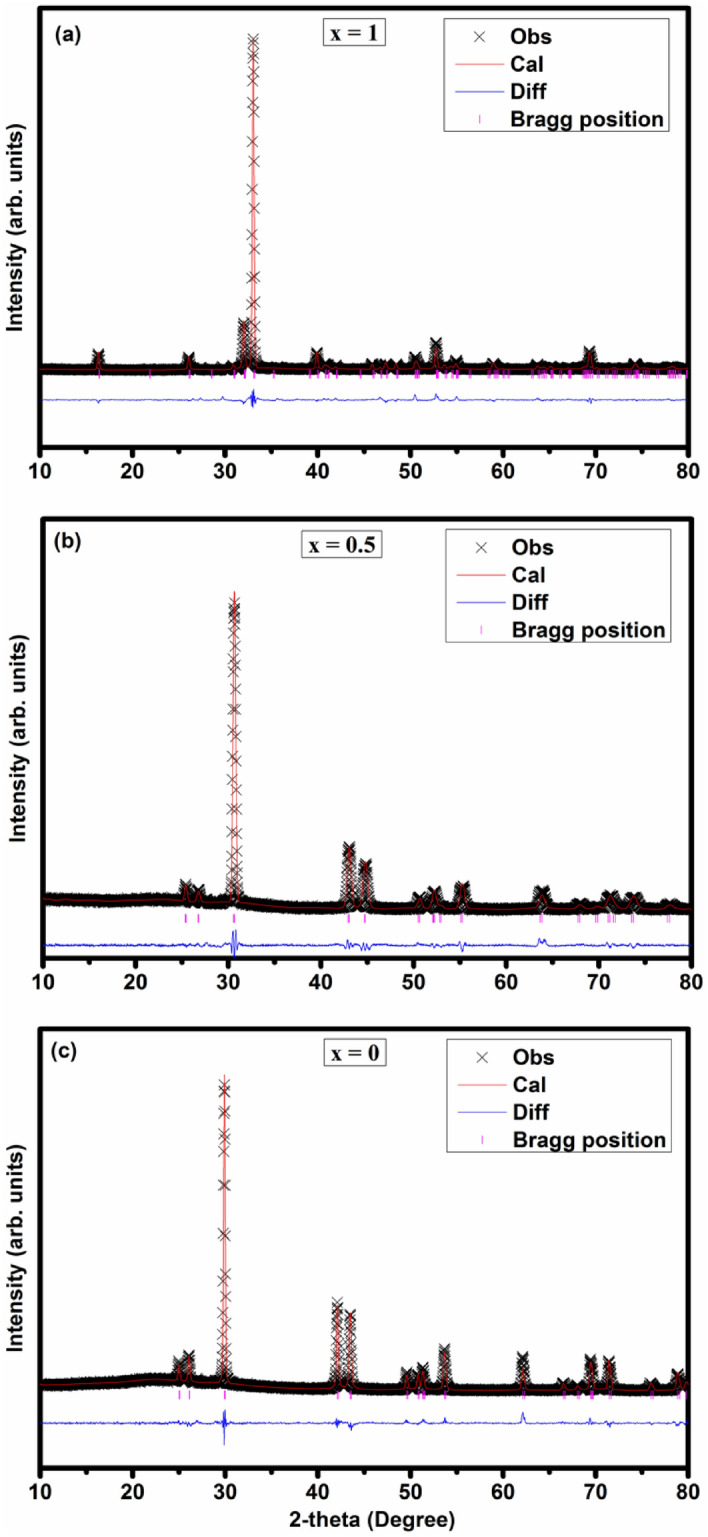
Rietveld Refined data of representative GeTe_1−x_Se_x_ samples (x = 0, 0.5 and 1.0) to verify structural transformation.

**Figure 3:**
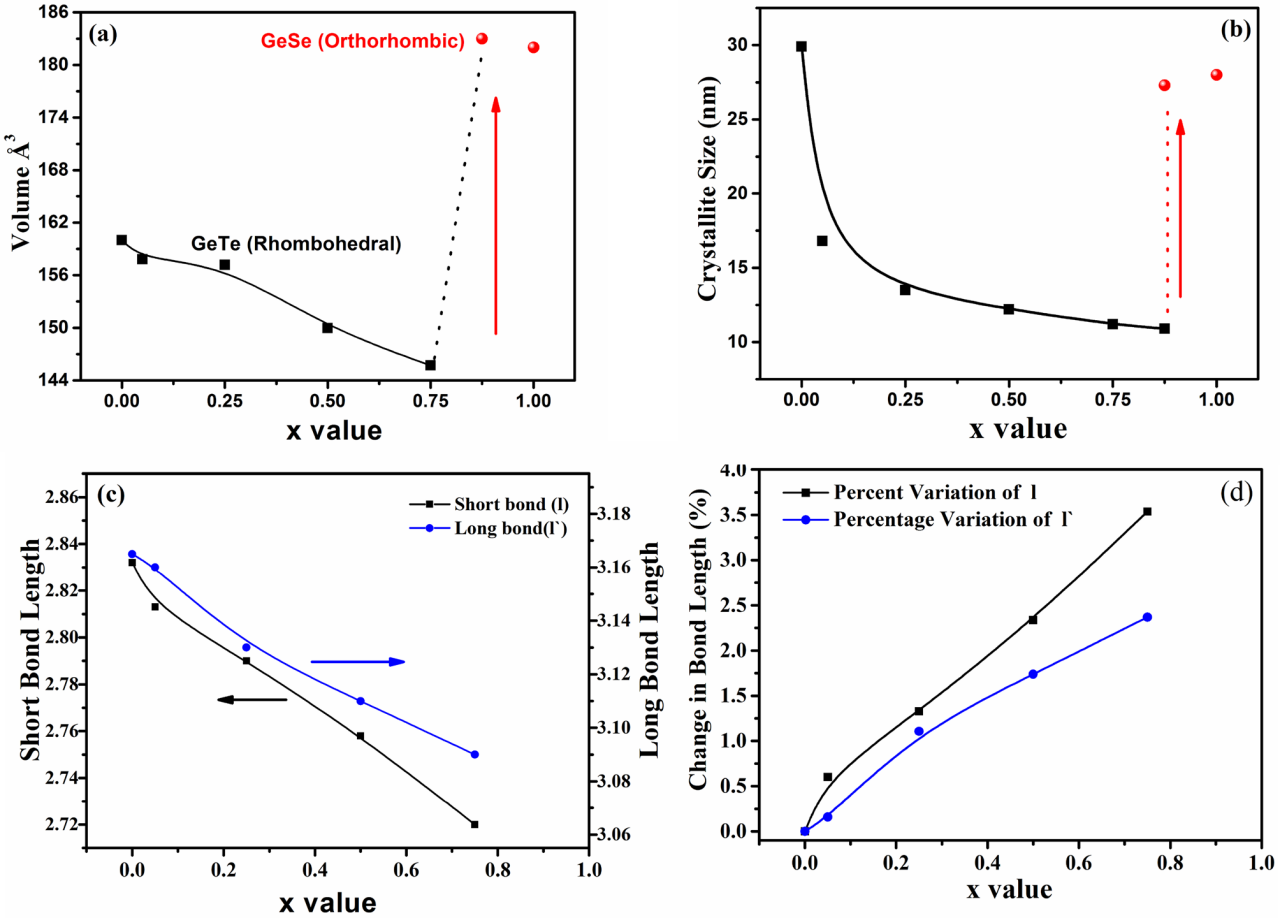
(**a**) Volume and (**b**) crystallite size of the GeTe_1−x_Se_x_ bulk samples determined from Rietveld data showing a large change when the layer structured GeSe starts dominating the structure at x ≥ 0.875. The red spheres represent for x = 0.875 and 1.0 where the influence of GeSe on the structure is becoming more pronounced. (**c**) Short and long bond length with Se substitution is showing the decrease of the length of both bonds. (**d**) Change in bond length percentage with Se substitution is found higher in short bonds compared to long bonds.

Structure of the Se substituted samples were further analyzed through Rietveld refinement using GeTe as a reference structure for x ≤ 0.75 samples. Calculated and observed refinement patterns for samples x = 0, 0.5 and 1.0 shown in Fig. 2 with the difference profile are in good agreement with experimentally measured pattern. XRD pattern of x = 0.875 has the mixture of two phases. Thus for this sample, multi-phase refinement has been followed. The estimated parameters are enlisted in Table 1. There is a systematic reduction in lattice parameters, crystallite size and volume of GeTe_1−x_Se_x_ samples for x ≤ 0.75 as shown in Fig. 3. For x > 0.75 sample, lattice parameters and volume of unit cells show higher values due GeSe orthorhombic phase formation. The increase in the FWHM of the peaks indicates a reduction in coherently scattering domains. It can be correlated with the local distortion created by Se in the GeTe structure due to its smaller atomic size of Se.

**Table 1:** Rietveld refinement data of GeTe_1−x_Se_x_ alloys.

Chemical formula	GeTe	x = 0.01	x = 0.25	x = 0.5	x = 0.75	x = 0.875		GeSe
Crystal System	Rhomb	Rhomb	Rhomb	Rhomb	Rhomb	Rhomb/ Ortho		Ortho
Space group	R 3 m H	R 3 m H	R 3 m H	R 3 m H	R 3 m H	R 3 m H/ Pcmn		Pcmn
**Unit cell parameters (Å)**								
a	4.15960	4.14441	4.12928	4.05591	4.01731	4.3640		4.38793
b	4.15960	4.14441	4.12928	4.05591	4.01731	3.85586		3.83275
c	10.6770	10.6087	10.6431	10.5258	10.4293	10.8768		10.8182
c/a	2.5668	2.5699	2.5774	2.5951	2.5961	N/A		N/A
Unit cell volume (Å^3^)	159.98640	157.80559	157.16297	149.95558	145.71	182.99		181.94
R values								
R_wp_	0.082	0.059	0.0666	0.0734	0.1341	0.0697		0.0858
R F_2_	0.0803	0.0419	0.0431	0.0601	0.2197	0.0895		0.1048
X^2^	1.998	1.993	1.536	2.188	1.277	3.408		2.28
Crystallite size (nm)	29.9	16.8	13.5	12.2	11.2	10.9	27.3	28

To understand the structural changes in PCM materials two parameters are considered to be important, viz. ionicity (r_σ′_) and hybridization (r_π_^−1^)^21^. The parameter r_σ′_ is determined by the electronegativity difference of p-orbitals of two nearby atoms and r_π_^−1^ is the quantification of covalency. The rhombohedral structure of GeTe is characterized by low r_σ′_ and low r_π_^−1^ parameter in the r_σ′_ vs. r_π_^−1^ map^21^. In contrast, GeSe is located at a comparatively high r_σ′_ and r_π_^−1^ values. Based on the r_σ′_ and r_π_^−1^, the binary IV–VI group materials are grouped into three categories, i.e., cubic, rhombohedral, and orthorhombic^21^. Cubic to rhombohedral transition occurs due to transverse optic phonons coupling with electrons from partially filled p-orbitals of chalcogenides in [111] direction and is mainly characterized by r_σ′_ and is independent of the r_π_^−1^. Increasing of this parameter drives the system towards a more symmetric structure. Cubic to orthorhombic transition is assisted by the variation of r_π_^−1^ parameter (due to hybridization of s-p orbitals) of involved atoms with no significant variation in the r_σ′_ parameter. The effect of the high value of r_π_^−1^ is that the material tends to form a layered structure and bond firmly with atoms in the same layer and bond loosely with atoms in the adjacent layers. However, the transition from rhombohedral to orthorhombic structure can be understood by considering the changes in the atomic arrangement in short and medium-range order with increasing Se. Rhombohedral and orthorhombic structures share the same type of short-range order, i.e., three-fold co-ordinated pyramidal structures interacting with nearest neighbors (NN) in layered form. The difference in these structures appears from the variation of the medium-range order and depends on the next-nearest neighbor (NNN) position. The rhombohedral phase is a slightly distorted form of the cubic structure along [111] direction concerning to the central atom. It causes the formation of a layered structure perpendicular to [111] direction. This distortion causes six equidistant neighbors of cubic structure to split up into two categories, i.e., NN (atoms within the same layer) forming a strong bond with length (l) and NNN (atoms forming comparatively longer bonds of length (l′) with atoms in parallel layers). Thus, these structures have nearly the same NN configuration, but the difference mainly arises from the NNN positions.

GeTe shows two types of bonds in the rhombohedral structure, i.e., short bond at 2.83 Å and long bond at 3.16 Å. The substitution of smaller Se atoms for larger Te atoms in GeTe result reduction of the length of both the short and long bonds as seen from the Rietveld refinement (Fig. 3c). But, a closer look at the percentage variation of the length of the long and short bonds reveal that the variation in the length of the long bonds is less compared to short bonds for x ≤ 0.75 (Fig. 3d). It is a signature of increased inter-planar spacing due to increased r_π_^−1^ in Se substituted samples. When Se is replacing any of the Te atoms, Ge forms a short and strong bond with the Se atom owing to the high electronegativity of Se. As a result, the strength of the longer bond decreases. With increasing Se, this trend continues and the NN bonds keep on getting strengthened, while the longer bonds become weaker. When the Ge–Se bonds exceed a critical concentration, it starts losing the interlayer contact completely and advances towards forming a separate layered GeSe phase. In GeTe_1−x_Se_x_ samples, this critical Se concentration is found to occur at x = 0.75. Thus for GeSe (total substitution of Te by Se), a fully layered structure forms, where Ge and Se bond firmly with its 3 NN while, the NNN lies in the next layer and is connected only through van der Waal interaction. The variation in short bond length is higher due to strong covalent bonds forming in the same layer. This can be seen from Fig. 3d, exhibiting a higher slope for the percentage change in short bond length. Experimentally, we observe less variation in bond length along z-axis of the lattice.

To study the phase change properties of same materials, thin films of Se substituted samples were prepared from thermal evaporation method using bulk samples as starting material. As deposited films were found to be amorphous in nature. Upon annealing at high temperatures, GeTe films showed a gradual change from amorphous to cubic NaCl phase.

**Figure 4:**
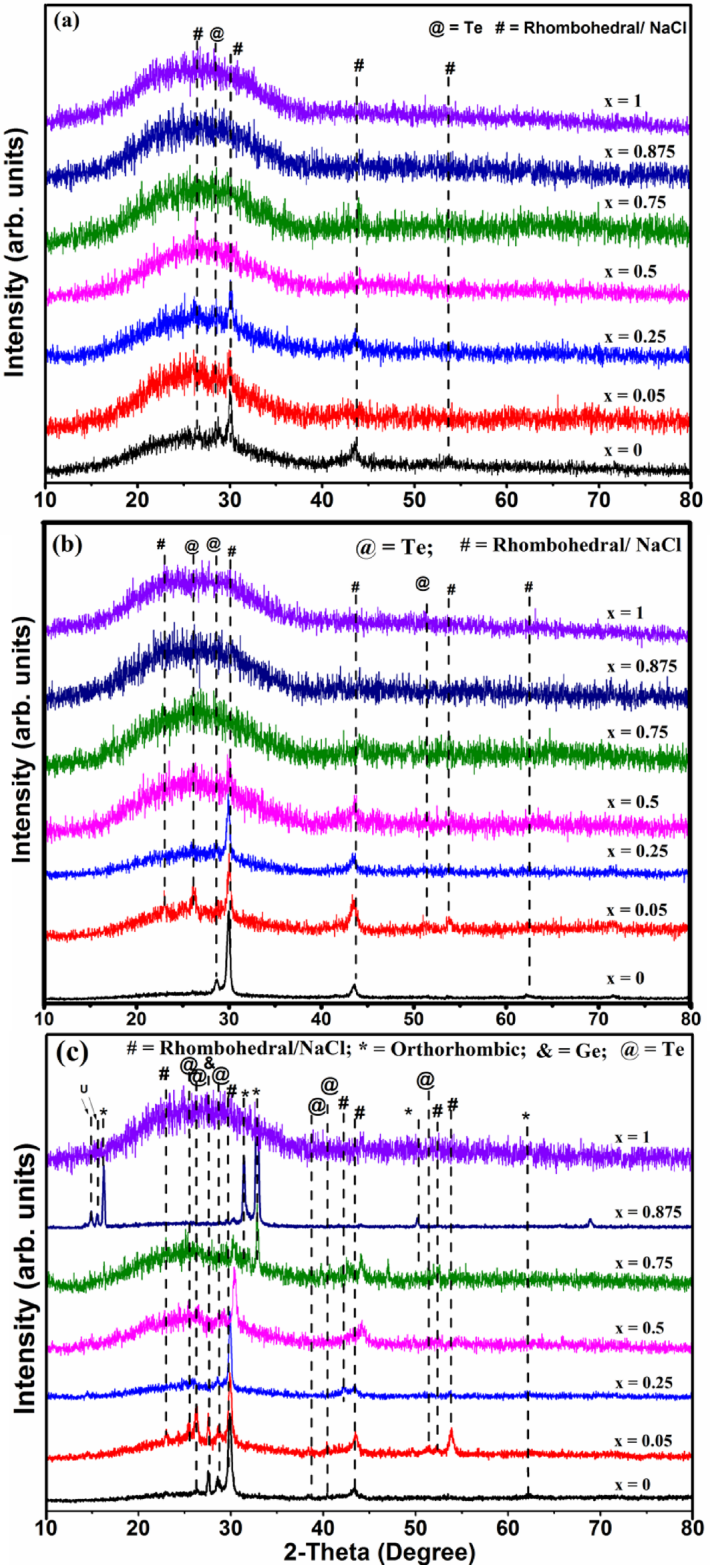
XRD of the samples annealed at (**a**) 150, (**b**) 200 and (**c**) 300 °C showing the evolution of the crystalline phases.

**Figure 5:**
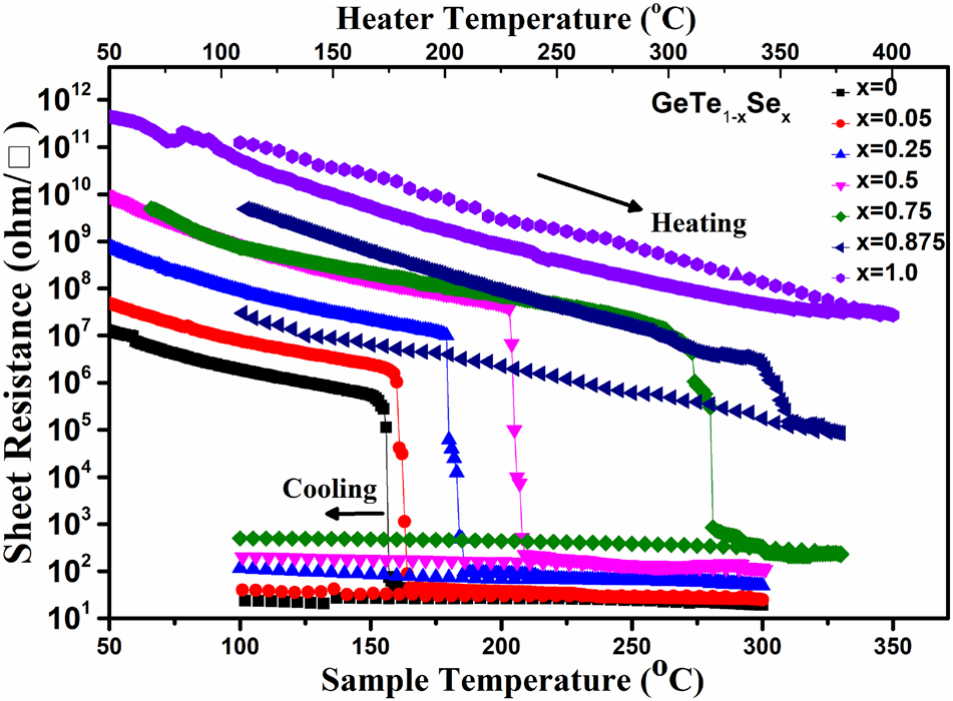
In situ Sheet Resistance vs Temperature measurement of Ge_1_Te_1−x_Se_x_ films, showing phase transition. Both sample and heater temperatures are shown for reference. The sample temperature is measured by keeping thermo-couple on top of sample. Heater temperature is measured on the top of the heater surface.

XRD patterns of the GeTe_1−x_Se_x_ films annealed at 150 °C, 200 °C and 300 °C temperatures are shown in Fig. 4 with their corresponding phases. The film with x = 0 shows crystallization at 150 °C (Fig. 4a) which also closely agrees with transition temperature observed in R-T measurement (Fig. 5). Crystallization is also observed for films x = 0.05 and 0.25 but the observed intensity is lower compare to the intensity of the XRD peaks of x = 0. For these samples the main crystalline phases observed are NaCl phase, with Te as a secondary phase^25^. All remaining samples are amorphous at this temperature. It is interesting to note that with a small addition of Se films annealed at 150 °C are amorphous owing to the easy glass formability of the additive Se^26^.

When GeTe_1−x_Se_x_ films annealed at 200 °C (Fig. 4b), crystallization is observed for x ≤ 0.5. However, the intensity of the alloy with x = 0.5 is found to be less compared to the alloy with x = 0.05 and 0.25. It demonstrates that Se substitution hinders the crystallization of GeTe and increases the crystallization temperature and therefore the thermal stability of the amorphous phase. It should also be mentioned that there were no Se based segregates found, as evident in the XRD of the annealed films. Annealing of the GeTe_1−x_Se_x_ films at 300 °C resulted in crystallization of all samples (0 ≤ x ≤ 0.875) except for x = 1.0 as shown in Fig. 4c. The samples 0.25 ≤ x ≤ 0.75 crystallize into rhombohedral phase instead of NaCl phase as in x = 0 and 0.05. For x = 0.75, secondary GeSe phase peaks found to start appear (Fig. 4c).

In-situ resistance vs. temperature (R-T) measurements were carried out on the as-deposited amorphous films to understand the amorphous to crystalline phase transition. Fig. 5 shows the R-T curves for GeTe_1−x_Se_x_ films. Initially, resistance shows a continuous decrease and at T_c_, the resistance shows an abrupt drop, indicating an amorphous to crystalline transition. The initial reduction in resistance with increasing temperature can be understood by thermally activated hopping transportation^27^ of charge carriers from the valence band to the conduction band. The transition from amorphous to the crystalline state in samples x ≤ 0.5 is in a single step with no second phase transition. This transition corresponds to amorphous to the NaCl crystal structure for x = 0 and x = 0.05. For 0.25 ≤ x ≤ 0.5 the observed transition is from amorphous to rhombohedral, which also reflects the XRD results. An abrupt drop of resistance in these samples at T_c_ indicates that the crystallization speed is high. As soon the crystalline nuclei are formed, crystal growth would be almost instantaneous and provide a contrast in resistance of 4 order or higher. Fig. 5 shows the shift of the transition temperature to the higher side with the increase of Se supporting our previous findings.

The transition temperature for x = 0 agrees well with the values reported in the literature^28,29^. According to the report by Coombs et al.^30^, for Ge_50_Te_50−x_Se_x_ samples, with increasing Se, growth time remains nearly constant but nucleation time increases drastically. This leads to the rise in the time taken by Se substituted samples to form critical nuclei. Hence for Se substituted samples, T_c_ shifts to higher temperature value. As the growth time is nearly unaffected, thus once the nuclei form, crystal growth takes place almost instantaneously in the sample and give the sharp drop in transition. This trend is followed in the samples upto x = 0.5. For x = 0.75 also, we observe the trend followed but with the second transition. The first transition for this sample correspond to the amorphous to orthorhombic (GeSe) phase transition and second transition correspond to amorphous to rhombohedral (as evident from XRD data of corresponding thin films). Additionally, the contrast in electrical resistance between the amorphous and crystalline phases of the GeTe_1−x_Se_x_ samples increases with Se concentration. It is interesting to note that for x = 0.5 (50% at Te sites), the contrast in resistance is about 6 orders of magnitude. This shows the contrast in resistance of GeTe can be tuned to a larger extent by Se substitution at Te sites. Also note that Se substitution increases the resistance of the crystallized (SET) phases (Fig. 5), which is beneficial for lowering the power requirements of the PCM devices^31^. While cooling the sample, the resistance showing a small increase in resistance indicating that the crystallized samples are also semiconducting in nature. From Fig. 5, it is observed that the RESET resistance of Se substituted samples is higher than the GeTe, which can facilitate in more extensive data read margin and lower RESET current^8^ in a device. Hence, Se substitution enhances the T_c_, resistance contrast, and lowers the RESET power.

As expected, for x = 1.0 film, there is no significant change in resistance with increasing temperature. Therefore, GeSe may not be useful as a PCM material. Based on the above results, it can be suggested that up to 50% Se substitution (x = 0.5) in GeTe_1−x_Se_x_, is beneficial for memory applications.

**Figure 6:**
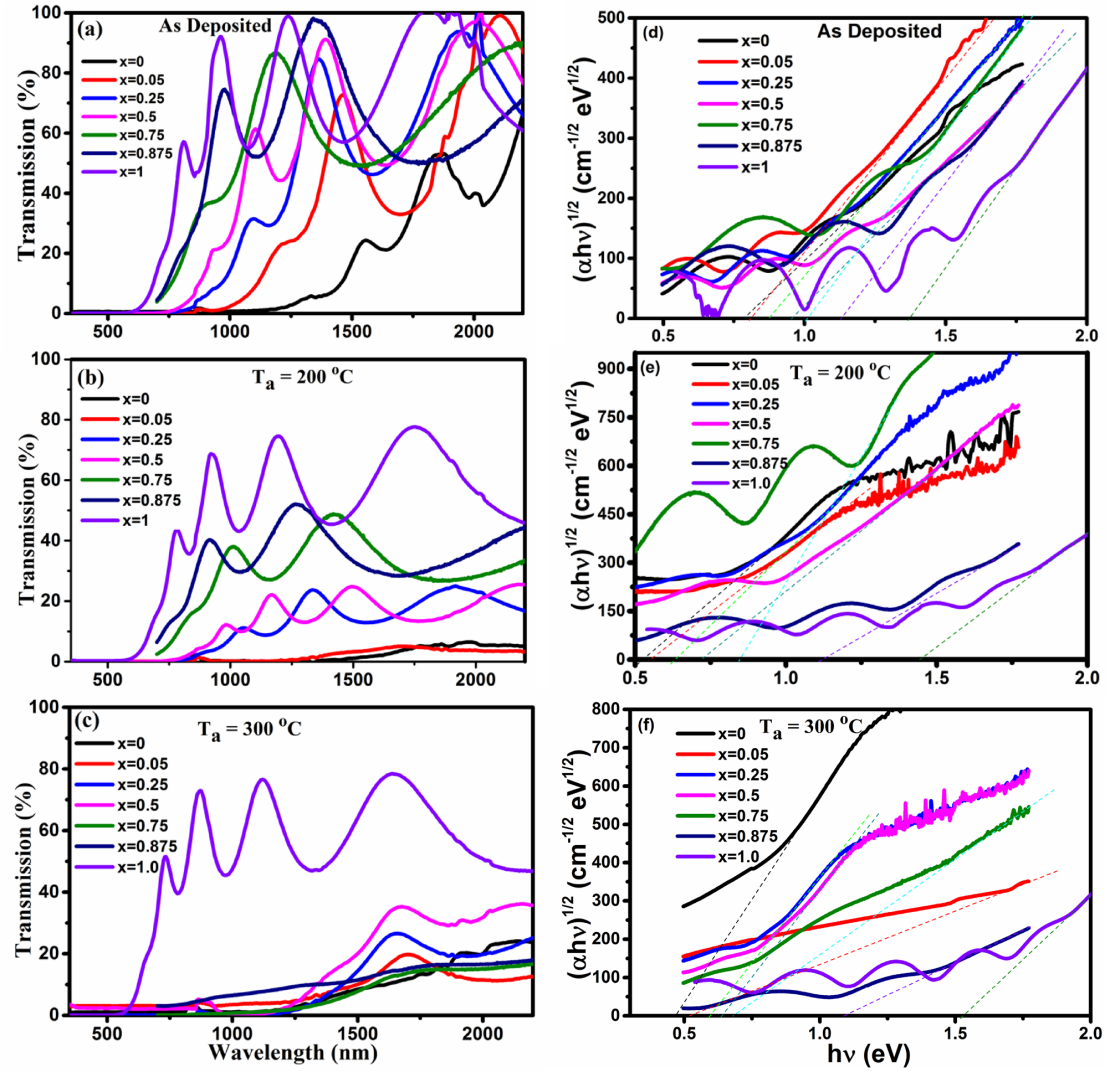
Transmission spectra of the GeTe_1−x_Se_x_ films (**a**) as deposited and annealed films (**b**) at 200 °C and (**c**) at 300 °C. The corresponding Tauc plot are shown in (**d**)–(**f**).

Optical UV–Vis-NIR transmission spectra of the as deposited and annealed GeTe_1−x_Se_x_ samples are shown in Fig. 6a–c. The absorption edge of the as deposited x = 0 films is 1200 nm, and gradually shifts to higher energy with the increase of Se substitution. The absorption edge observed for x = 1.0 is 600 nm. Annealing of the x = 0 and x = 0.05 films at 200 °C reduces the transmittance maxima considerably, indicating crystallization of these alloys, which is in line with the XRD results (Fig. 4). When annealed at 200 °C, the absorption edge shifted to 1500 nm for x = 0, and 600 nm for x = 0.05. When the annealing temperature is increasing to 300 °C, the transmittance of the film (0 ≤ x ≤ 0.875) decrease to a larger extend with a corresponding shift in their absorption edge. This could be due to the increase in the crystallinity of the films. In all the cases, the absorption edge of x = 1.0 remains at 600 nm, and there is no significant changes in the transmittance spectra, indicating that GeSe remains amorphous and does not undergo phase change till 300 °C.

The Tauc plot used for calculating the band gap of GeTe_1−x_Se_x_ films is shown in Fig. 6d–f. For higher values (α > 10^4^ cm^−1^), the absorption coefficient α yields the power part which obeys the Tauc^32^ and Davis and Mott relation^33,34^ for the allowed indirect transition:

1 $$\left( {\alpha {\text{h}}\nu } \right)^{{{1}/{2}}} = {\text{ B}}^{{{1}/{2}}} \left( {{\text{h}}\nu \, - {\text{ E}}_{{{\text{opt}}}} } \right)$$

where B^1/2^ is the Tauc parameter, h is the Plank's constant, υ is the frequency and E_opt_ is the optical band gap. The band gap of as deposited films varies between 0.78 eV for x = 0 to 1.37 eV for x = 1.0 indicating that the band gap increases with the Se substitution. The films annealed at 200 °C show a reduction in band gap compared to the as deposited films. For single crystal GeSe (x = 1.0), the indirect band gap is reported to be 1.14 eV^35^. For Ge_25_Se_75_ glass the band gap is around 1.78 eV and for Ge_40_Se_60_, the band gap is 1.58 eV^36^. For x = 1.0, we have obtained an indirect band gap of 1.37 eV.

**Figure 7:**
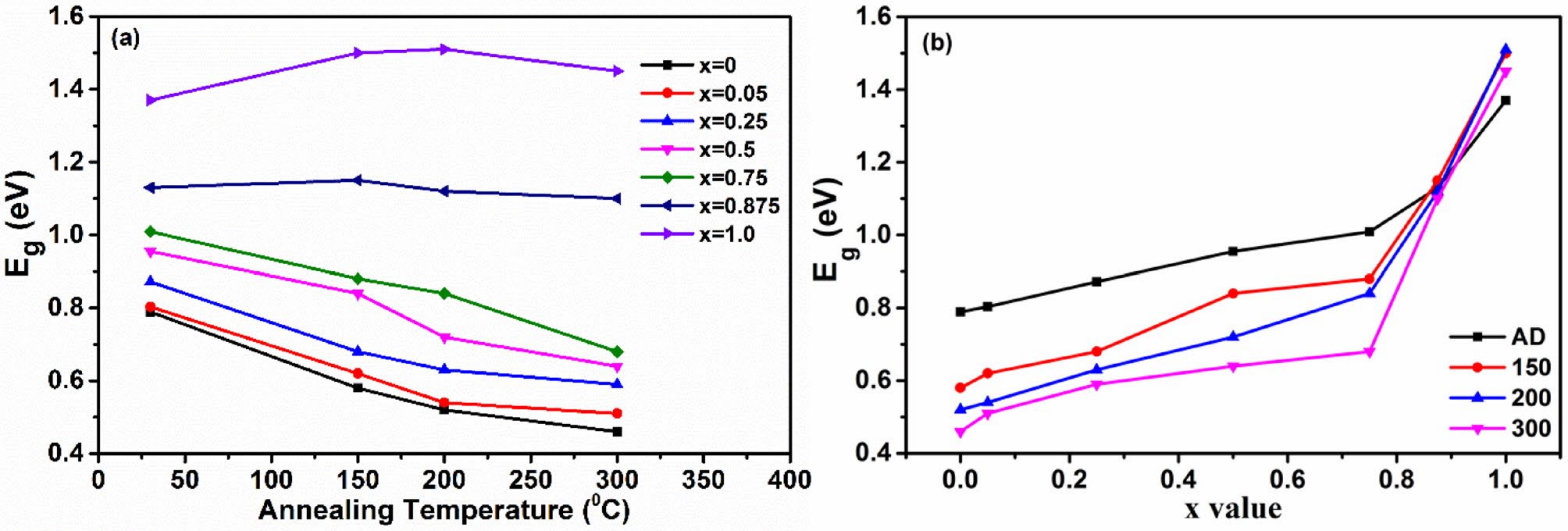
(**a**) Bandgap as a function of annealing temperature. (**b**) Bandgap variation with Se content for different annealing temperatures.

 Fig. 7 shows the band gap of GeTe_1−x_Se_x_ samples as a function of T_a_ and Se content. The optical contrast in band gap values between its amorphous and crystalline states can be seen in Fig. 7a. In the case of x = 1.0, the variation in the band gap is insignificant, which verifies this material does not possess phase contrast. From Fig. 7b, it can be seen that the calculated band gap is increasing with increasing Se content signifying the possibility of tuning the band gap with Se substitution. The band gap shows a jump at x = 0.75, indicating a change in Te dominated structure to a Se dominated structure. The rate of increase of band gap is relatively high for x > 0.75. This may be related to the change in the nucleation dominated crystallization for x = 0 to growth dominated structures for x = 1.0^26^.

As the Se content increases in the alloy, the preference for bonding changes. The higher energy Ge–Se bonds form at the expense of low energy Ge–Te bonds^37^. Qualitatively, x = 0.05 in GeTe_1−x_Se_x_ (5% of Se at Te sites) leads to 5% of Ge–Se bonds. The remaining Ge bonds with Te to form the GeTe phase in the as deposited films. When annealed, the films with x = 0 crystallizes first which is also very well seen from the XRD results. The GeSe phase needs higher energy to crystallize^6^, and hence they remain amorphous. This continues for higher Se substituted alloys as well. In the case of x ≥ 0.75 sample, more number of Ge–Se than Ge–Te bonds would be probable. GeSe phase is seen in 300 °C annealed films along with the GeTe phase (Fig. 4c). A detailed study may be required to understand more about the role of GeSe in the phase change behavior of GeTe_1−x_Se_x_ alloys.

When the as deposited films were annealed above T_c_, the structure transforms from NaCl/Rhombohedral to orthorhombicdue to hybridization of s-p orbitals of involved atoms. When s to p hybridization energy becomes smaller, they form sp hybridized orbitals, and a resilient covalent structure becomes viable. Stronger s-p hybridization increases the gap between bonding and anti-bonding states and wide open the forbidden band gap. Hence, with the increase of Se, there is an increase in the band gap.

I-V characteristics define the behavior of the device under the influence of an applied electrical field. Phase change memory (PCM) materials switches between their amorphous and crystalline states upon the application of a current pulse. The contrast in electrical resistance between these states is significant. Primarily, the electro-thermal mechanism is used to explain the switching effect in chalcogenide glassy semiconductors. This includes the thermal effects due to the Joule heating and electronic contribution due to the applied field in switching the materials from amorphous to the crystalline state. This model considers a significant role of defect states that trap the charge carriers. The conduction is through hopping and tilting potential regions (Poole–Frenkel conduction). This property is utilized in the PCRAM applications. Initially the current and voltage are linear (ohmic) and for a critical voltage called threshold voltage (V_th_), it exhibits a non-linear (non-ohmic) behavior leading to the negative differential resistance region. This leads to the abrupt increase in the current resulting structural phase transition from a low conducting amorphous (OFF) state to a high conducting crystalline (ON) state. In PCM materials, the device state (ON/OFF) is preserved even after removing the applied electric field.

**Figure 8:**
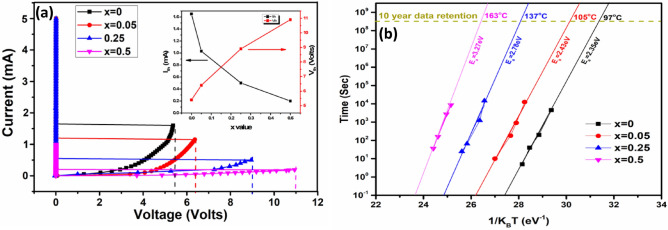
(**a**) I–V characteristics of GeTe_1−x_Se_x_ films. The inset shows the dependence of I_th_ and V_th_ on the Se concentration. (**b**) Data Retention measurement for corresponding samples.

In GeTe_1−x_Se_x_ samples, a memory switching is observed for 0 ≤ x ≤ 0.5 (Fig. 8a). Samples with x > 0.5 does not show switching. Formation of a filament in between the electrodes due to the Joule heating is responsible for the memory switching. This filament consists of high conducting crystallites. GeTe switched to the high conducting state at a threshold voltage of 5.4 V, and the corresponding threshold current (I_th_) is 1.65 mA. The measured V_th_ for Se substituted samples with x = 0.05, 0.25, 0.5 are 6.4 V, 8.9 V, 10.9 V and the corresponding I_th_ values are 1.03 mA, 0.5 mA, and 0.2 mA respectively. V_th_ shows an increasing trend while the I_th_ showed a decreasing trend with the increase of Se (see the inset in Fig. 8a). This increasing value of V_th_ is the consequence of the formation of high energy bonds in the Se substituted samples. A high value of V_th_ and low value of the corresponding I_th_ of Se substituted GeTe samples, make sure that the device consumes a low power via the relation P = V_th_*I_th_. Additionally, Low thermal conductivity of the GeSe phase makes sure the power confinement in the limited region leads to low power consumption^16^. The data retention values were calculated using Arhenius equation^18^ and shown in Fig. 8b. The activation energies (and 10-year data retention temperatures) for x = 0, 0.05, 0.25 and 0.5 are found to be 2.35 eV (97 °C), 2.43 eV (105 °C), 2.78 eV (137 °C) and 3.27 eV (163 °C), respectively. With increasing Se substitution in GeTe, data retention increases which is beneficial for PCM applications.

### Conclusions

In this study, We have proposed ternary compositions GeTe_1−x_Se_x_ (0 ≤ x ≤ 1.0) along the pseudo-binary tie line between GeTe-GeSe, for high temperature phase change memory applications. Structure of bulk samples studied on the basis of ionicity and hybridization concept, and it has been observed that the Se substituted GeTe samples mainly form rhombohedral lattice, but with increasing content of Se, distortions increases. Above a certain concentration (x > 0.75), segregation begin to occur and GeSe phase appears. From XRD measurements on the corresponding thin films, it has been observed that Se subtituted samples show no secondary phase separation for x ≤ 0.5, and two phases for x > 0.5. The x = 0.5 sample uniquily shows both, a largest resistance contrast and higher Tc compared to other Se substituted alloys. The resistance contrast between the amorphous and the crystalline phase for x = 0.5 is about 6 orders of magnitude, which make it better than the undoped GeTe. The band gap and the Vis–NIR transmittance varies due to Se substitution as a result of the structural modifications and possibly due to the formation of higher energy Ge–Se bonds. With the Se substitution, the band gap increased in the as deposited films and decreased in the annealed films. Results shows Se substitution can effectively be used to tune the bandgap and transition temperatures of GeTe systems. I-V switching and data retention measurements has also shown promising results for x = 0.5 sample. Based on this data, it can be concluded that the substitution of Se, up to 50% (x = 0.5) in GeTe_1−x_Se_x_ is beneficial for memory applications.

## Methods

Bulk GeTe_1−x_Se_x_ (0 ≤ x ≤ 1.0) alloys were prepared by melt quenching method. Appropriate amounts of highly pure (5 N) Ge, Te, and Se were weighed and sealed in quartz ampoules under a vacuum of 10^–6^ mbar. Ampoules containing the raw materials were loaded into a rocking furnace. The furnace temperature was increased from RT to 950 °C with the rate of 100 °C/hr and kept for 72 h. The furnace was rocked frequently during this period to homogenize the melt. The ampoules containing alloy melt were quenched into the ice-cold water. The synthesized bulk sample were collected from the ampoule and ground in an agate mortar. The bulk powders were subjected to XRD to understand its crystal structure. GeTe_1−x_Se_x_ thin films of 500 nm thickness were prepared by the thermal evaporation method. The powder samples were used as the starting material for deposition. A sigma SQC-310 deposition controller unit was used for controlling deposition at a deposition rate of 5 Å/s in a base vacuum of 5 × 10^–6^ mbar. During the deposition, at normal incidence, the substrates were suitably rotated to obtain a uniform thickness. Elemental compositions of the alloys were verified by Energy-dispersive X-ray spectroscopy (EDS) and the results are shown in Table 2. Results of all the bulk powder and thin films are provided below with ± 3% error.

**Table 2:** EDS data for bulk powder and thin films are enlisted in the table.

Sample name	Elements	Expected (at.%)	Bulk (at.%)	Thin film (at.%)
GeTe	Ge	50	54.86	51.14
	Te	50	45.14	48.86
GeTe_0.95_Se_0.05_	Ge	50	52.74	53.65
	Te	47.5	44.35	44.31
	Se	2.5	2.91	2.04
GeTe_0.75_Se_0.25_	Ge	50	52.99	50.77
	Te	37.5	33.04	33.49
	Se	12.5	13.97	15.74
GeTe_0.5_Se_0.5_	Ge	50	49.48	49.31
	Te	25	23.86	26.88
	Se	25	26.66	23.81
GeTe_0.25_Se_0.75_	Ge	50	49.94	49.20
	Te	12.5	12.38	11.88
	Se	37.5	37.68	38.92
GeTe_0.125_Se_0.875_	Ge	50	50.89	47.1
	Te	6.25	7.43	7.4
	Se	43.75	41.68	45.5
GeSe	Ge	50	48.87	46.99
	Se	50	51.13	53.01

Thicknesses were confirmed by the Stylus Profilometer (DEKTAK VEECO) and were annealed at 150, 200, and 300 °C for 2 h to study the phase formation and structural behavior. X-ray diffraction (XRD) patterns were recorded using Bragg–Brentano geometry at room temperature employing a BRUKER D8 ADVANCE with CuK_α_ radiation (λ = 1.54 Å) at 40 kV and 30 mA. Data collection was performed with a continuous scan from 2-theta 10º to 80º in step of 0.02^o^ at a scan speed of 2º/min. The powder data found matching with the standard reference pattern of GeTe on ICSD with reference number 56038^19^, space group R3mH and GeSe with reference number 637863^38^, space group Pcmn. Structure of the GeTe and the Se substituted GeTe samples i.e. GeTe_0.95_Se_0.05_, GeTe_0.75_Se_0.25_, GeTe_0.5_Se_0.5,_ GeTe_0.25_Se_0.75_ and GeTe_0.125_Se_0.875_ were refined along GeSe sample by using Rietveld Refinement analysis. The refinement was carried out using the GSAS software suite using a graphical user interface EXPGUI. The starting structure for refinement is Rhombohedral GeTe. For diffraction profile modeling Pseudo-Voigt function was utilized. In GeSe sample a strong orientation in [004] direction was found which makes peak (004) most intense, due to preferred orientation, which is treated using March-Dollas function in GSAS. TEM measurements were performed using Titan Themis 300 kV from FEI instrument. Recorded Selected Area Electron Diffraction (SAED) patterns were analysed using Gatan GMS3 software.

Resistance vs. Temperature measurements were carried out using a custom-made probe station under a vacuum of 2 × 10^–3^ mbar in the temperature range of 50–400 °C. Temperatures of the sample and heater were measured simultaneously by keeping thermocouples (K-type) on top of the sample and heater surfaces. A two probe configuration was used to measure in situ resistance values at a 3 °C/min heating rate using Keithley Electrometer Model 6517-b. Each resistance reading was an average of three measurements. Two probe resistance measurements were carried out using the same Agilent Device Analyzer on the samples annealed at different temperatures. Transmission spectra of the films were taken using Perkin Elmer Lambda 750 UV–VIS–NIR Spectrometer in the range (250–2250 nm). For electrical switching studies, devices in a sandwich geometry of Al/PCM/Al (Area:2 mm and thickness:300/500/300 nm) on glass substrates were fabricated. The current was swept between upper and bottom Al electrodes, and the corresponding voltages were measured using Agilent DC probe station 2 (PMS, Agilent Device Analyzer B1500A with pulsed source 5 MHz).
